# Prevalence and determinants of fever, diarrhea, and acute respiratory infection among children aged 5–59 months in Somaliland, 2020: insights from a nationwide survey

**DOI:** 10.3389/fped.2026.1811275

**Published:** 2026-05-04

**Authors:** Abdilaahi Yusuf Nuh

**Affiliations:** 1 School of Postgraduate Studies and Research, University of Burao, Burao, Somalia; 2 College of Medicine and Health Sciences, University of Burao, Burao, Somalia

**Keywords:** acute respiratory infection, childhood illnesses, determinants, diarrhea, fever, Somali demographic and health survey

## Abstract

**Background:**

Acute respiratory infections (ARIs), diarrhea, and fever remain major public health burdens among children in low- and middle-income countries, including Somaliland. These illnesses contribute substantially to childhood morbidity and healthcare utilization. This study aimed to determine the prevalence and determinants of these common childhood illnesses in Somaliland.

**Methods:**

Secondary analysis of data from the 2020 Somali Demographic and Health Survey (SDHS), a nationwide cross-sectional survey, was conducted. The analysis focused on children aged 5–59 months. Data on diarrhea, fever, and acute respiratory infection (ARI) in the two weeks preceding the survey were analyzed using binary logistic regression to identify associated factors.

**Results:**

A total of 4,702 children aged 5–59 months were included in the analysis. The prevalence of fever, diarrhea, and ARI was 6.07%, 4.75%, and 3.66%, respectively. Child age was the most consistent determinant: children aged ≥25 months had significantly lower odds of diarrhea (AOR = 0.53; 95% CI: 0.31–0.90), fever (AOR = 0.53; 95% CI: 0.31–0.90), and ARI (AOR = 0.37; 95% CI: 0.20–0.67) compared to those aged ≤12 months. Notable regional variations were identified; children residing in Togdheer demonstrated substantially lower odds of diarrhea (AOR = 0.30; 95% CI: 0.14–0.67; *p* = 0.003), fever (AOR = 0.30; 95% CI: 0.14–0.68; *p* = 0.004), and ARI (AOR = 0.29; 95% CI: 0.12–0.71; *p* = 0.006) in comparison to those in Maroodi-Jeeh. A nomadic lifestyle was associated with decreased odds of diarrhea (AOR = 0.48; 95% CI: 0.31–0.75; *p* = 0.001), fever (AOR = 0.49; 95% CI: 0.32–0.77; *p* = 0.002), and ARI (AOR = 0.58; 95% CI: 0.34–0.98; *p* = 0.042) relative to rural living. Access to healthcare emerged as a significant protective factor against diarrhea (AOR = 0.43; 95% CI: 0.28–0.67; *p* < 0.001) and fever (AOR = 0.44; 95% CI: 0.28–0.68; *p* < 0.001), though not for ARI (AOR = 0.81; 95% CI: 0.52–1.27; *p* = 0.363). Vaccination status did not show a significant association with diarrhea (AOR = 1.12; 95% CI: 0.58–2.16; *p* = 0.726), fever (AOR = 1.12; 95% CI: 0.58–2.17; *p* = 0.727), or ARI (AOR = 1.37; 95% CI: 0.65–2.89; *p* = 0.401).

**Conclusions:**

Childhood illnesses remain a public health concern in Somaliland, particularly among younger children. Key determinants include child age, maternal age, geographic location, residence type, and access to healthcare. Targeted interventions focusing on early childhood, improving healthcare access, and addressing regional disparities are essential to reduce morbidity.

## Introduction

Acute respiratory infections (ARIs), diarrheal diseases, and febrile illnesses remain major contributors to childhood morbidity in low- and middle-income countries (LMICs). Although substantial progress has been made in reducing under-five mortality globally, these conditions continue to impose a high burden of disease, particularly among children under five years of age. ARIs and diarrheal diseases are among the leading causes of pediatric illness episodes, contributing to repeated healthcare utilization, impaired nutritional status, and increased vulnerability to subsequent infections (1, 2).

The occurrence of these illnesses is influenced by a complex interaction of socioeconomic, environmental, and maternal factors. Poor sanitation, unsafe drinking water, inadequate hygiene practices, low maternal education, limited access to healthcare services, and household crowding have all been consistently associated with increased risk of diarrhea and respiratory infections among children (3, 4). In addition, suboptimal breastfeeding practices and incomplete immunization coverage further increase susceptibility to infectious diseases during early childhood (5, 6).

Efforts to reduce childhood morbidity are central to global health priorities, particularly under Sustainable Development Goal (SDG) 3, which aims to ensure healthy lives and promote well-being for all ages. Despite commitments to global health systems, progress toward universal health coverage remains uneven across sub-Saharan Africa and the Horn of Africa, where fragile health systems, environmental challenges, and socioeconomic inequalities continue to hinder improvements in child health outcomes (7). In Somalia and Somaliland, recurrent drought, poverty, and limited healthcare infrastructure contribute to persistent exposure to preventable childhood illnesses, particularly in rural and nomadic populations (8).

In Somaliland, evidence on the epidemiology of diarrhea, fever, and ARIs remains limited and often aggregated at the national level, masking important regional and contextual differences. Understanding how these illnesses vary by sociodemographic and environmental characteristics is essential for designing targeted interventions and improving child health outcomes. Therefore, this study utilized data from the Somali Health and Demographic Survey 2020 to estimate the prevalence of diarrhea, fever, and ARIs among children aged 5–59 months in Somaliland and to identify key determinants associated with these conditions. The findings are expected to inform context-specific public health strategies aimed at reducing childhood morbidity and improving healthcare access in the region.

## Method and materials

### Study setting

This study was conducted in Somaliland, a self-governing region in the Horn of Africa. For this study, and in accordance with the SDHS, Somaliland is treated as a geographic region included within the national survey framework. Somaliland is administratively divided into six regions: Awdal, Maroodi-Jeeh, Sahil, Togdheer, Sanaag, and Sool. The region covers approximately 176,120 square kilometers and had an estimated population of 5.7 million in 2021 (9).

### Study design

This study employed a cross-sectional design using data from the SDHS 2020, the most recent nationally representative dataset available. The analysis focused on children aged 5–59 months residing in Somaliland.

### Data limitations

This analysis relies on maternal self-reports of illness in the two weeks preceding the survey, which may be subject to recall bias and misclassification. The mobility of nomadic households may result in the underrepresentation of certain groups. Due to the cross-sectional nature of the SDHS, causal inferences cannot be drawn.

### Source of data

This study used data from the 2020 Somali Demographic and Health Survey (SDHS), a nationally representative household survey conducted by the Somali National Bureau of Statistics in collaboration with the Ministry of Health of Somalia (8). The survey collected demographic, socioeconomic, and health-related information using standardized DHS questionnaires and procedures.

For this analysis, records were restricted to households located in Somaliland. Regional and district identifiers were used to isolate observations from the six Somaliland regions. As the SDHS is the only recent nationally representative survey that includes Somaliland, the SDHS 2020 dataset provides the most reliable data source for examining childhood illness patterns in this context.

After filtering for the six regions and applying the appropriate DHS sampling weights, the analytical sample represents children aged 5–59 months residing in Somaliland. In the raw SDHS dataset, some districts currently belonging to the Sahil region were coded under the former Waqooyi-Galbeed region. These districts (Berbera and Sheikh) were recoded to Sahil using district identifiers to align the data with the present administrative boundaries of Somaliland. The remaining districts were classified under Maroodi-Jeeh. This ensured accurate representation of the six Somaliland regions: Awdal, Maroodi-Jeeh, Sahil, Togdheer, Sanaag, and Sool (10).

### Data extraction

The children's recode (KR) file from the SDHS 2020 was used. The analytical sample was restricted to children aged 5–59 months who were living with their mothers at the time of the survey and had complete information on diarrhea, fever, and acute respiratory infection (ARI) within the two weeks preceding the survey. Children aged 0–4 months were excluded because DHS illness questions (fever, diarrhea, ARI) have lower reliability in neonatal and young-infant age groups, and symptom recognition is substantially different from that in older infants. Additionally, the distribution of ARI symptoms is biologically distinct in the first months of life; therefore, restricting analysis to 5–59 months aligns with prior DHS analytical practices, and records with missing or “don't know” responses for outcome or key explanatory variables were excluded. Sampling weights provided by SDHS were applied to account for the complex survey design and ensure nationally representative estimates for Somaliland.

### Sampling and sample size

The SDHS 2020 employed a three-stage stratified cluster sampling design. Each administrative region was stratified into urban, rural, and nomadic categories. In the initial stage, Primary Sampling Units (PSUs) were selected using probability proportional to size. In the subsequent stage, Secondary Sampling Units (SSUs) were selected within urban and rural PSUs. However, due to the mobile nature of the population, nomadic PSUs did not use the SSU stage; instead, households were listed directly within the selected PSU. In the SDHS sampling design, the nomadic stratum is treated as a distinct residence category separate from both rural and urban areas. Nomadic households are sampled independently and do not fall under either urban or rural classifications. In the final stage, households were systematically selected from the household listing within each PSU. Approximately 30 households were selected from each PSU, and all eligible women aged 15–49 years were interviewed.

The study population comprised women aged 15–49 years who were either permanent residents or visitors present in selected households on the night preceding the survey. A total of 13,210 women completed the interview process. For this study, the sample was restricted to mothers of children aged 5–59 months who provided comprehensive information regarding their child's illness history, including fever, acute respiratory infections (ARI), and diarrhea. Following the application of inclusion criteria and sampling weights, the dataset used for analysis comprised children aged 5–59 months with complete information on all study variables. Only complete cases with non-missing data for all variables of interest were included in the multivariable analyses.

A formal power calculation was not conducted for this secondary analysis due to the utilization of the fixed SDHS 2020 dataset. Nevertheless, the final analytical sample of 4,702 children is deemed adequate for multivariable logistic regression, in accordance with recommended guidelines that necessitate at least 10 outcome events per predictor variable. The number of illness cases in the dataset surpassed this requirement, indicating sufficient statistical power.

In the Somali Demographic and Health Survey (SDHS) 2020, a total of 13,210 women were interviewed. From the children's recode file, 15,628 children aged 0–59 months were identified. After excluding 1,335 children who were outside the eligible age range of 5–59 months, 14,293 children remained. Further exclusions were made due to missing illness information (*n* = 5,200) and missing covariate data (*n* = 4,391). All children were residing with their mothers at the time of the survey. After applying eligibility criteria and excluding records with missing data, the analytical dataset was derived from children aged 5–59 months with complete information on all study variables Supplementary A.

### Variables

#### Outcome variables

Three childhood illnesses were examined as dependent variables in this study: diarrhea, fever, and acute respiratory infection (ARI). Each outcome was defined according to the Demographic and Health Survey (DHS) standard criteria.

Diarrhea was defined as the mother/caregiver reporting that the child experienced three or more loose or watery stools within 24 h during the two weeks preceding the survey. Fever was defined as a maternal/caregiver report of fever occurring in the two weeks before the survey. ARI was defined as a reported episode of cough accompanied by short, rapid, or difficult breathing related to a chest problem in the two weeks preceding the survey.

Each outcome variable was coded as a binary variable (1 = illness present, 0 = illness absent) and was analyzed separately using multivariable logistic regression. No composite outcome variable was constructed.

#### Independent variables

The selection of independent variables was guided by a conceptual framework informed by the socio-ecological model of child health and prior Demographic and Health Survey (DHS)-based studies on childhood morbidity in low- and middle-income countries (3, 11, 12). Childhood illnesses such as diarrhea, fever, and ARI are influenced by multiple interacting factors at the child, maternal, household, and community levels. Accordingly, variables including child age and sex, maternal age and education, household wealth index, place of residence, region, water source, and access to health services were included. These variables were selected based on their established associations with childhood illness in previous DHS analyses and their relevance to pathways involving exposure to infection, caregiving practices, environmental sanitation, and healthcare utilization. Table 1 summarizes these variables and their operational definitions.

**Table 1:** Independent variables and operational definitions.

Variable	Operational definition
Region	Awdal, Maroodi-Jeeh, Sahil, Togdheer, Sanaag, Sool
Child Age	Categorized as 5–12, 13–24, 25–59 months
Sex of Child	Male/Female
Maternal Age	15–24, 25–34, ≥35 years
Maternal Education	None, Primary, Secondary, Higher
Wealth Index	DHS quintiles: Lowest → Highest
Household Size	Number of household members
Residence Type	Urban, Rural, Nomadic
Drinking Water Source	Improved/Unimproved
Distance to Health Facility	Big problem/Not a big problem
Vaccination Status	Fully vaccinated/Not fully vaccinated

A conceptual framework illustrating the relationships between these variables and childhood illnesses is presented in Supplementary B.

### Statistical methods

Data analysis was conducted using STATA version 17. In order to accommodate the intricate sampling design of the SDHS, the analysis incorporated sampling weights, clustering, and stratification variables. The DHS sampling weight (v005) was divided by 1,000,000, as recommended by DHS guidelines, and applied using survey (svy) commands in STATA. This adjustment ensures that prevalence estimates and regression results accurately represent the target population and that standard errors appropriately account for the multi-stage cluster sampling design. All analyses were conducted using the STATA “svy” commands to account for the complex sampling design of the SDHS. Sampling weights were applied, and clustering and stratification were adjusted to produce nationally representative estimates. Both crude and adjusted analyses incorporated these survey design features to ensure valid standard errors and confidence intervals.

Descriptive statistics were used to summarize the prevalence of childhood illnesses [fever, acute respiratory infection (ARI), and diarrhea] among children aged 5–59 months. Bivariate analyses employing cross-tabulations and Pearson's chi-square (*χ*²) tests were performed to assess associations between each disease (fever, ARI, diarrhea) and sociodemographic, environmental, and maternal-child health variables.

Variables associated with each outcome at *p* < 0.25 in the bivariate analysis were considered for inclusion in the multivariable logistic regression models. This relatively liberal threshold was applied to minimize the risk of excluding potential confounders that may not show strong unadjusted associations but are important in adjusted models. This approach is consistent with widely accepted recommendations for the purposeful selection of variables in regression modeling (13, 14). **Statistical significance was set at *p* < 0.05, with levels indicated as follows: *p < 0.05, **p < 0.01, and ***p < 0.001.

Multicollinearity among independent variables was assessed using Variance Inflation Factors (VIF). The mean VIF was 1.84, with values ranging from 1.12 to 3.76, indicating no evidence of significant multicollinearity among the explanatory variables (all VIF values < 10). The methodological framework for logistic regression aligns with established practices, as described by Cramer (2002) (15).

## Result

### Prevalence of common childhood illnesses

**Figure 1:**
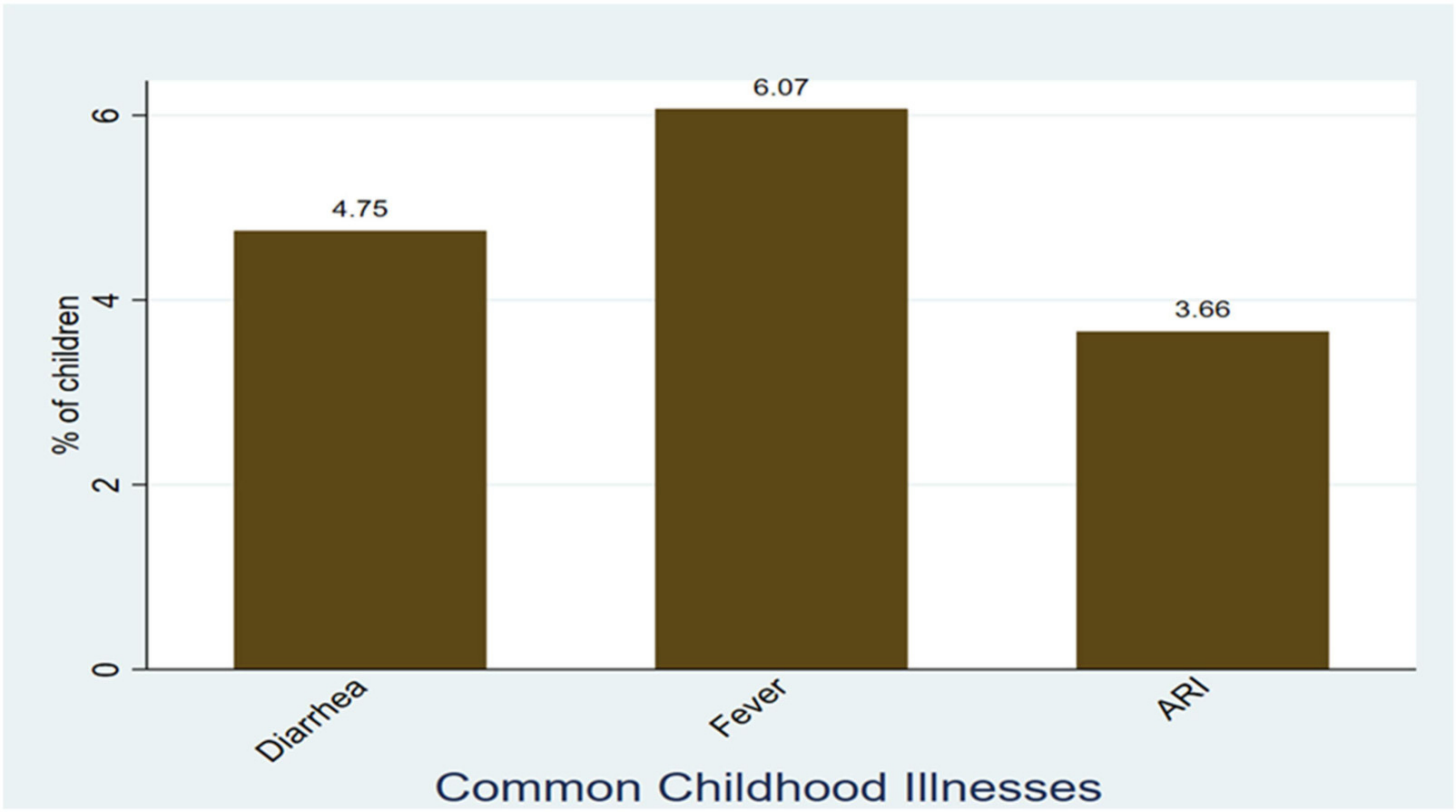
Weighted prevalence of childhood illnesses among children aged 5–59 months in Somaliland, SDHS 2020.

The study evaluated the prevalence and determinants of common childhood illnesses in Somaliland, specifically diarrhea, fever, and acute respiratory infections (ARI), among the 4,702 children aged 5–59 months included in the analysis. The most frequently reported illness was fever, affecting 6.07% (95% CI: 5.3–6.9) of the children, followed by diarrhea at 4.75% (95% CI: 4.1–5.4), and acute respiratory infection (ARI) at 3.66% (95% CI: 3.1–4.3) (Figure 1).

### Determinants and distribution percentage of childhood illnesses

As shown in Table 2. The age of the child emerged as a significant determinant of diarrhea incidence (*p* < 0.001). Children aged 13–24 months exhibited the highest prevalence (7.89%), followed by those under 12 months (6.86%), while children aged 25 months or older had the lowest prevalence (3.40%). This pattern is consistent with increased exposure to contaminated food and environments during the weaning period.

**Table 2:** Sociodemographic, environment, and health characteristics of studied children aged 5–59 months and their percentage distribution by diarrhea, fever, and acute respiratory infection (ARI) in somaliland, 2020 (*n* = 4,702).

Variables	Categories	Diarrhea (%)		*P*-value	Fever (%)		*P*-value	ARI (%)		*P*-value
		Yes	No		Yes	No		Yes	No	
Sex of child	Male	5.07	94.93	0.532	6.13	93.87	0.869	4.00	96.00	0.253
	Female	4.66	95.34		6.19	93.81		3.68	96.32	
Age of child (in months)	<12	6.86	93.14	<0.001***	9.33	90.67	<0.001***	5.99	94.01	<0.001***
	13–24	7.89	92.11		8.68	91.32		4.60	95.40	
	25–59	3.40	96.60		4.64	95.36		2.86	97.14	
Breastfeeding Status	Ever breastfed (not currently)	4.34	95.66	<0.001***	5.58	94.42	<0.001*	3.55	96.45	0.097
	Never Breastfed	2.84	97.16		3.33	96.67		2.46	97.54	
	Still breastfeeding	7.60	92.40		9.71	90.29		4.76	95.24	
Vaccination Status	No	4.63	95.37	0.033*	6.04	93.96	0.034*	4.14	95.86	0.013*
	Yes	7.94	92.06		9.72	90.28		7.80	92.20	
Maternal Age	15–19	1.78	98.22	0.009**	6.51	93.49	0.039*	4.71	95.29	0.6551
	20–24	5.80	94.20		7.80	92.20		3.65	96.35	
	25–29	5.92	94.08		5.10	94.90		3.30	96.70	
	30–34	4.24	95.76		5.41	94.59		3.65	96.35	
	35–39	5.16	94.84		6.46	93.54		3.47	96.53	
	40–44	6.20	93.80		6.25	93.75		4.90	95.10	
	45–49	1.10	98.90		12.36	87.64		6.45	93.55	
Maternal educational level	No Education	4.64	95.36	0.2134	5.72	94.28	0.004**	3.69	96.31	0.521
	Primary	6.12	93.88		9.28	90.72		4.05	95.95	
	Secondary	7.29	92.71		6.06	93.94		1.01	98.99	
	Higher	2.04	97.96		2.08	97.92		4.08	95.92	
Wealth index	Lowest	5.80	94.20	0.693	5.76	94.24	0.723	3.31	96.69	0.810
	Second	6.14	93.86		6.10	93.90		4.27	95.73	
	Middle	5.50	94.50		5.55	94.45		3.31	96.69	
	Fourth	6.51	93.49		6.40	93.60		3.86	96.14	
	Highest	7.08	92.92		92.85	7.15		3.52	96.48	
Region	Awdal	5.62	94.38	<0.001***	6.71	93.29	0.006**	2.88	97.12	0.023*
	Maroodi-Jeeh	4.29	95.71		8.13	91.87		4.71	95.29	
	Saahil	11.18	88.82		9.15	90.85		4.87	95.13	
	Togdheer	2.33	97.67		4.49	95.51		1.87	98.13	
	Sool	4.80	95.20		6.00	94.00		4.50	95.50	
	Sanaag	3.68	96.32		6.71	93.29		3.39	96.61	
Place of residence	Rural	6.52	93.48	<0.001***	7.99	92.01	<0.001***	4.68	95.32	0.036*
	Urban	5.64	94.36		6.31	93.69		3.45	96.55	
	Nomadic	3.01	96.99		4.59	95.41		3.00	97.00	
Distance to a health facility	Big problem	5.24	94.76	0.117	6.81	93.19	0.016	3.59	96.41	0.631
	Not a big problem	4.14	95.86		4.94	95.06		3.88	96.12	
Source of drinking water	Improved	5.07	94.93	0.50	6.03	93.97	0.90	3.56	96.44	0.86
	Unimproved	4.61	95.39		6.12	93.88		3.66	96.34	

* Significant association *p* < 0.05.

** Significant association *p* < 0.01.

*** Significant association *p* < 0.001.

Breastfeeding status showed significant variation in diarrhea prevalence (*p* < 0.001), with children who were still breastfeeding having a higher reported prevalence (7.60%) compared to those never breastfed (2.84%). Similarly, vaccinated children showed a higher prevalence of diarrhea (7.94%) compared to unvaccinated children (4.63%) (*p* = 0.033).

Regional disparities were evident, with Saahil region reporting the highest diarrhea prevalence (11.18%, *p* < 0.001). Place of residence was also significant, with higher prevalence in rural areas (6.52%) compared to nomadic settings (3.01%) (*p* < 0.001). Maternal age showed a statistical association (*p* = 0.009), although the pattern was not consistent across all categories.

Fever prevalence was highest among children aged <12 months (9.33%), followed by those aged 13–24 months (8.68%, *p* < 0.001). Vaccinated children again showed higher reported fever prevalence (9.72%) compared to unvaccinated children (6.04%, *p* = 0.034), which likely reflects similar reporting and healthcare access biases rather than a causal relationship. Maternal education was significantly associated with fever (*p* = 0.005), with the highest prevalence among children of mothers with primary education (9.28%). Regional variation persisted, with Saahil reporting elevated rates (9.15%, *p* = 0.006). Fever prevalence was also higher in rural areas (7.99%) compared to urban (6.31%) and nomadic settings (4.59%) (*p* < 0.001). Interestingly, children living closer to health facilities reported lower fever prevalence (4.94%, *p* = 0.016), possibly reflecting better access to early treatment.

The prevalence of acute respiratory infection (ARI) was highest among children aged 13–24 months (4.60%, *p* < 0.001). Vaccinated children had a higher reported prevalence (7.80%) than unvaccinated children (4.14%, *p* = 0.013); however, as noted above, this likely reflects confounding and differences in healthcare utilization rather than a true causal effect. Significant regional variation was observed (*p* = 0.023), with Togdheer reporting the lowest prevalence (1.87%). ARI prevalence was higher in rural areas (4.68%) compared to urban (3.45%) and nomadic populations (3.00%) (*p* = 0.036).

### Associated factors with diarrhea, fever, and acute respiratory infection (ARI)

Associated factors with diarrhea, fever, and acute respiratory infection (ARI): The results should be presented in the text following the same sequence as the factors listed in Table 3.

**Table 3:** Survey-weighted multivariable logistic regression analysis of factors associated with diarrhea, fever, and acute respiratory infection (ARI) among children in Somaliland, 2020.

Variable/category	Diarrhea AOR (95% CI)	*p*	Fever AOR (95% CI)	*p*	ARI AOR (95% CI)	*p*
Maternal age						
20–24^a^	–	–	–	–	–	–
15–19	0.70 (0.31–1.56)	0.381	0.70 (0.31–1.56)	0.383	1.12 (0.46–2.72)	0.808
25–29	0.62 (0.39–0.99)	0.043	0.61 (0.38–0.97)	0.036	0.64 (0.35–1.18)	0.154
30–34	0.50 (0.30–0.85)	0.011	0.50 (0.30–0.85)	0.011	1.00 (0.55–1.81)	0.998
35–39	0.60 (0.35–1.04)	0.069	0.60 (0.35–1.04)	0.071	0.94 (0.49–1.79)	0.848
40–44	0.31 (0.09–1.05)	0.060	0.31 (0.09–1.04)	0.058	1.79 (0.74–4.36)	0.200
45–49	2.17 (0.88–5.34)	0.092	2.16 (0.88–5.32)	0.094	2.14 (0.69–6.63)	0.188
Region						
Maroodi-Jeeh^a^						
Awdal	0.80 (0.36–1.76)	0.577	0.80 (0.36–1.75)	0.569	0.31 (0.11–0.90)	0.031
Saahil	0.71 (0.33–1.53)	0.379	0.70 (0.33–1.52)	0.371	0.67 (0.29–1.56)	0.351
Togdheer	0.30 (0.14–0.67)	0.003	0.30 (0.14–0.68)	0.004	0.29 (0.12–0.71)	0.006
Sool	0.65 (0.33–1.28)	0.216	0.64 (0.33–1.26)	0.196	0.58 (0.28–1.22)	0.150
Sanaag	0.50 (0.25–1.00)	0.050	0.50 (0.25–1.00)	0.051	0.44 (0.20–0.95)	0.037
Age category						
>12^a^	–	–	–	–	–	–
13–24	1.04 (0.65–1.67)	0.857	1.03 (0.64–1.65)	0.900	0.64 (0.37–1.13)	0.122
25–59	0.53 (0.31–0.90)	0.019	0.53 (0.31–0.90)	0.019	0.37 (0.20–0.67)	0.001
Vaccination status						
No^a^	–	–	–	–	–	–
Yes	1.12 (0.58–2.16)	0.726	1.12 (0.58–2.17)	0.727	1.37 (0.65–2.89)	0.401
Breastfeeding status						
Ever breastfed (not currently)	–	–	–	–	–	–
Never breastfed	0.39 (0.15–0.97)	0.043	0.40 (0.16–1.01)	0.051	0.35 (0.13–0.99)	0.048
Still breastfeeding	1.21 (0.79–1.86)	0.379	1.23 (0.80–1.89)	0.345	0.61 (0.36–1.03)	0.065
Residence						
Rural^a^	–	–	–	–	–	–
Urban	0.67 (0.41–1.10)	0.112	0.68 (0.41–1.11)	0.125	0.98 (0.57–1.68)	0.943
Nomadic	0.48 (0.31–0.75)	0.001	0.49 (0.32–0.77)	0.002	0.58 (0.34–0.98)	0.042
Distance to Health Facility						
Big problem^a^	–	–	–	–	–	–
Not a big problem	0.43 (0.28–0.67)	<0.001	0.44 (0.28–0.68)	<0.001	0.81 (0.52–1.27)	0.363
Constant	0.41 (0.16–1.02)	0.054	0.40 (0.16–1.01)	0.054	0.29 (0.10–0.83)	0.021

a Reference category: AOR, Adjusted Odds Ratio; CI, Confidence Interval. Reference categories indicated. Model adjusted for age category, region, residence, breastfeeding status, and vaccination status. Statistically significant results at *p* < 0.05.

The maternal age was associated with the incidence of diarrhea and fever. Offspring of mothers aged 25–29 years (AOR = 0.62; *p* = 0.043) and 30–34 years (AOR = 0.50; *p* = 0.011) demonstrated lower odds of diarrhea, with similar findings for fever, while no significant association was observed for ARI. Additionally, Children's age was significantly correlated with all three health outcomes. Compared to children aged ≤12 months, those aged 13–24 months showed no significant differences, whereas children aged 25–59 months exhibited reduced odds of experiencing diarrhea (AOR = 0.53; 95% CI: 0.31–0.90; *p* = 0.019), fever (AOR = 0.53; 95% CI: 0.31–0.90; *p* = 0.019), and ARI (AOR = 0.37; 95% CI: 0.20–0.67; *p* = 0.001).

Regional differences were significant. Children residing in Togdheer had reduced odds of diarrhea (AOR = 0.30; *p* = 0.003), fever (AOR = 0.30; *p* = 0.004), and ARI (AOR = 0.29; *p* = 0.006) compared to those in Maroodi-Jeeh. Furthermore, lower odds of ARI were noted in Awdal and Sanaag. The type of residence was associated with morbidity. Compared to rural households, nomadic households exhibited lower odds of diarrhea (AOR = 0.48; *p* = 0.001), fever (AOR = 0.49; *p* = 0.002), and ARI (AOR = 0.58; *p* = 0.042), whereas urban residence did not show significant associations.

The distance to a health facility was significantly associated with diarrhea and fever; children whose caregivers reported that distance was not a significant issue had lower odds of diarrhea (AOR = 0.43; *p* < 0.001) and fever (AOR = 0.44; *p* < 0.001), but not ARI. Additionally, breastfeeding status exhibited a partial association. Children who were never breastfed had lower odds of diarrhea (AOR = 0.39; *p* = 0.043) and ARI (AOR = 0.35; *p* = 0.048), while vaccination status was not significantly associated with any of the outcomes.

## Discussion

This study provides region-specific evidence on the prevalence and determinants of diarrhea, fever, and acute respiratory infection among children aged 5–59 months in Somaliland using nationally representative survey data. The observed prevalence of diarrhea (4.75%), fever (6.07%), and ARI (3.66%) was lower than estimates reported in many sub-Saharan African settings, where diarrhea commonly ranges from 8% to 20% and ARI from 5% to 15% (12, 16). These differences may reflect variations in environmental conditions, timing of data collection, and potential under-reporting inherent in caregiver-reported survey data. Although the prevalence rates appear relatively low, they reflect short-term morbidity within a limited recall period and may be subject to recall and reporting bias. Therefore, these findings should be interpreted with caution, particularly in assessing broader population-level risk.

Child age emerged as a consistent determinant across all three illnesses, with children aged ≤12 months exhibiting the highest risk. This finding is consistent with previous studies indicating that infants are particularly vulnerable to infectious diseases due to immature immune systems and increased exposure to pathogens during the introduction of complementary feeding (2, 6). The significantly lower odds of illness among children aged 25–59 months likely reflect improved immune function and accumulated exposure to pathogens over time, a pattern widely documented in DHS-based studies (3).

Maternal age was significantly associated with diarrhea and fever, with children of younger mothers experiencing a higher risk. This is consistent with evidence suggesting that younger mothers may have limited caregiving experience, lower health literacy, and reduced access to health information, all of which can influence child health outcomes (3, 12). However, no significant association was observed between maternal age and ARI, indicating that respiratory infections may be more strongly influenced by environmental and household-level factors rather than maternal characteristics alone.

Geographic disparities were evident across regions, with children in certain areas experiencing significantly higher or lower odds of illness. Such spatial variation has been widely reported in sub-Saharan Africa and is often linked to differences in environmental exposure, sanitation infrastructure, access to healthcare, and population density (4). These findings highlight the need for geographically targeted interventions rather than uniform national strategies.

Interestingly, children from nomadic households reported a lower prevalence of diarrhea, fever, and ARI than rural populations. While this pattern may partly reflect reduced exposure to overcrowded environments and improved natural ventilation, these findings require cautious interpretation. Notably, both breastfeeding and vaccination status demonstrated statistically significant associations in the bivariate analysis. For breastfeeding, children who were still breastfeeding had a significantly higher prevalence of diarrhea and fever (*p* < 0.001). This should not be interpreted as breastfeeding increasing illness risk. Rather, younger children who are biologically more susceptible to infections are also the group most likely to be breastfed, resulting in an age-related confounding effect. This interpretation aligns with extensive evidence demonstrating that breastfeeding is protective against diarrhea, fever, and respiratory infections in early childhood (5, 6).

Similarly, vaccinated children exhibited a significantly higher prevalence of ARI (*p* = 0.013). This counterintuitive finding does not suggest any harmful effect of vaccination. Instead, it likely reflects care-seeking and reporting differences: families who complete vaccinations tend to have stronger engagement with the health system and are therefore more likely to recognize symptoms and report illnesses. In other words, families who adhere to health systems, such as vaccinating their children and continuing to breastfeed, are usually more aware of their children's health. This, in turn, may lead to a higher recorded infection rate, but not necessarily a higher actual infection rate. Comparable patterns have been described in DHS-based studies where healthcare-engaged households show higher reported morbidity due to increased interaction with healthcare services and improved symptom awareness (3, 12).

Also, lower reported morbidity among nomadic children may be influenced by under-reporting. Previous research indicates that nomadic and mobile populations may have limited access to healthcare, reduced interaction with formal health systems, and cultural differences in illness recognition and reporting behavior, which can lead to underestimation of disease prevalence (17). Therefore, interpretation of these findings must consider reporting bias, healthcare access, and contextual differences between population groups.

Access to healthcare services was significantly associated with reduced odds of diarrhea and fever. Children living in households where distance to a health facility was not considered a major problem were less likely to experience illness, highlighting the importance of geographic accessibility in promoting timely care-seeking and disease management (3). The lack of association with ARI may reflect differences in caregiver perception and treatment-seeking behavior for respiratory symptoms.

Overall, these findings underscore the importance of addressing both individual- and community-level determinants of childhood illness in Somaliland. Strengthening maternal education, improving access to safe water and sanitation, and enhancing access to healthcare, particularly in underserved and geographically disadvantaged areas, are critical to reducing the burden of preventable childhood diseases. Future studies should incorporate longitudinal designs and additional environmental and nutritional variables to understand causal pathways better and inform more effective interventions.

## Conclusion

This study presents nationally representative data on the prevalence and determinants of diarrhea, fever, and acute respiratory infections among children aged 5–59 months in Somaliland. While the overall prevalence of these illnesses was relatively low, several factors were significantly associated with morbidity, including child age, maternal age, region, type of residence, breastfeeding status, and perceived access to healthcare services.

Children in the first two years of life were identified as the most susceptible to illness, underscoring the necessity for enhanced preventive and clinical interventions during early childhood. The observed regional and residence-related disparities suggest that child health programs should account for geographic and population-specific conditions, particularly in rural and nomadic communities. Enhancing access to primary healthcare services, supporting younger mothers, and strengthening early childhood health programs may help reduce the burden of common childhood illnesses in Somaliland. Also, longitudinal studies are recommended to better understand causal relationships and to explore the mechanisms underlying regional and residence-related differences in childhood morbidity.

## Limitations

This study has several limitations;

- The cross-sectional design of the SDHS limits the ability to establish causal relationships between explanatory variables and childhood illnesses.
- Illness information was based on maternal recall of symptoms occurring within the previous two weeks, which may introduce recall bias or misclassification.
- Symptoms such as fever or cough may not always correspond to clinically confirmed diagnoses, potentially affecting measurement accuracy. Finally, although several important covariates were included in the analysis, residual confounding from unmeasured factors such as nutritional status, environmental exposures, or healthcare utilization patterns may remain.

## Data Availability

Publicly available datasets were analyzed in this study. This data can be found here: https://microdata.nbs.gov.so/index.php/catalog/50/get-microdata.
