# Supplementary material for: Prevalence and determinants of fever, diarrhea, and acute respiratory infection among children aged 5–59 months in Somaliland, 2020: insights from a nationwide survey
Source: Front Pediatr. 2026 May 4;14:1811275. doi: 10.3389/fped.2026.1811275 (PMC13180732; doi:10.3389/fped.2026.1811275)
Supplement: Supplementary file 1 [file Table1.docx]

Women interviewed SDHS 2020

n = 13,210

.

. count if living_with_mother==1

living_with_mother not found

r(111);

Children age 0–59 months

n = 15,628

.

. count if living_with_mother==1

living_with_mother not found

r(111);

Excluded

- Outside 5–59 months = 1,335

Children aged 5–59 months

n = 14,293

Excluded

- Not with mother = 0
- Missing illness = 5,200
- Missing covariates = 4,391

**Final sample**

**n = 4,702**

Figure 1: Flow chart showing the sample selection process for children aged 5–59 months included in the analysis, SDHS 2020.
