# Supplementary material for: Prevalence and determinants of fever, diarrhea, and acute respiratory infection among children aged 5–59 months in Somaliland, 2020: insights from a nationwide survey
Source: Front Pediatr. 2026 May 4;14:1811275. doi: 10.3389/fped.2026.1811275 (PMC13180732; doi:10.3389/fped.2026.1811275)
Supplement: Supplementary file 2 [file Table2.docx]

Figure 1: Conceptual Framework of Determinants of Childhood Illnesses.

Child Factors

- Age
- Sex

Childhood Illnesses

- Diarrhea
- Fever
- ARI

Maternal Factors

- Maternal Age
- Education

Household factors

- Wealth Index
- Water Source
- Household size

Community factors

- Residence
- Region
- Access to Health Facility
